# The Akt Inhibitor ISC-4 Synergizes with Cetuximab in 5-FU-Resistant Colon Cancer

**DOI:** 10.1371/journal.pone.0059380

**Published:** 2013-03-12

**Authors:** Joshua E. Allen, Jean-Nicolas Gallant, David T. Dicker, Shantu Amin, Rosalyn B. Irby, Arun K. Sharma, Wafik S. El-Deiry

**Affiliations:** 1 Laboratory of Translational Oncology and Experimental Cancer Therapeutics, Department of Medicine (Hematology/Oncology), Penn State Hershey Cancer Institute, Hershey, Pennsylvania, United States of America; 2 Department of Pharmacology, Penn State Hershey Cancer Institute, Hershey, Pennsylvania, United States of America; 3 Department of Medicine (Hematology/Oncology), Penn State Hershey Cancer Institute, Hershey, Pennsylvania, United States of America; Vanderbilt University Medical Center, United States of America

## Abstract

Phenylbutyl isoselenocyanate (ISC-4) is an Akt inhibitor with demonstrated preclinical efficacy against melanoma and colon cancer. In this study, we sought to improve the clinical utility of ISC-4 by identifying a synergistic combination with FDA-approved anti-cancer therapies, a relevant and appropriate disease setting for testing, and biomarkers of response. We tested the activity of ISC-4 and 19 FDA-approved anticancer agents, alone or in combination, against the SW480 and RKO human colon cancer cell lines. A synergistic interaction with cetuximab was identified and validated in a panel of additional colon cancer cell lines, as well as the kinetics of synergy. ISC-4 in combination with cetuximab synergistically reduced the viability of human colon cancer cells with wild-type but not mutant *KRAS* genes. Further analysis revealed that the combination therapy cooperatively decreased cell cycle progression, increased caspase-dependent apoptosis, and decreased phospho-Akt in responsive tumor cells. The synergism between ISC-4 and cetuximab was retained independently of acquired resistance to 5-FU in human colon cancer cells. The combination demonstrated synergistic anti-tumor effects *in vivo* without toxicity and in the face of resistance to 5-FU. These results suggest that combining ISC-4 and cetuximab should be explored in patients with 5-FU-resistant colon cancer harboring wild-type *KRAS*.

## Introduction

Phenylalkyl isoselenocyanates (ISCs), the isosteric selenium analogues of the naturally occurring isothiocyanate class of compounds [1], [2], were recently developed in our laboratories and have been shown to inhibit Akt activity [3], [4], [5], [6]. Among the investigated ISCs, ISC-4 has shown the best anti-tumor activity in preclinical models, both *in vitro* and *in vivo*, with promising safety [4]. ISC-4 has demonstrated modest preclinical efficacy against many tumor types *in vitro*, and anti-tumor effects have been reported in the context of melanoma when ISC-4 is used a mono-agent *in vivo* [4], [5], [6]. ISC-4 also has exhibited significant anti-tumor effects, via the inhibition of Akt, in colon cancer xenografts when used as a mono-agent and when used in combination with 5-fluorouracil (5-FU)—though the clinical utility of the combination was unclear [5].

The PI3K/Akt pathway is over-activated or altered in the majority of colorectal cancers, which is the third most frequently diagnosed and deadly cancer in the United States [7]. The standard-of-care chemotherapy for the advanced disease includes the FOLFOX, or FOLFIRI regimens, which are comprised of 5-FU, leucovorin, oxaliplatin, and irinotecan in addition to other treatment options including the anti-VEGF monoclonal antibody bevacizumab and the anti-EGFR monoclonal antibody cetuximab. While these treatment combination options have improved the survival of patients, additional non-toxic targeted treatment options are needed in the therapy-refractory setting of advanced colon cancer.

Though the preclinical mono-agent activity of ISC-4 is significant, combination therapy has not been thoroughly explored. We therefore searched for synergistic combinations with ISC-4 using a panel of FDA-approved drugs. Here, we report the identification of ISC-4 and cetuximab as a promising combination therapy that has synergistic anti-tumor activity *in vitro* and *in vivo* against human colon cancers harboring a wild-type *KRAS* gene.

## Materials and Methods

### Cell culture, cell viability assays, and reagents

Cell lines were obtained from ATCC and cultured in ATCC-recommended media in a humidified incubator at 5% CO_2_ and 37°C. Cell lines used in this study were not authenticated. For cell viability assays, cells were seeded into 96-well black-walled plates at a concentration of 1×10^5^ cells per mL in fresh media and in a volume of 100 µL per well. Cells were allowed to adhere overnight and were treated the next day as indicated. At endpoint, CellTiter-Glo (Promega) assays were performed according to the manufacturer's protocol, and the bioluminescent readout was recorded on an IVIS imaging system (Xenogen). For cell synchronization, cells were incubated with 200 ng/mL nocodazole for 16 hours prior to treatment. Chloroquine was obtained from Sigma. zVAD-fmk was obtained from Promega and used at a working concentration of 25 µM. ISC-4 was synthesized as previously described [6].

### Flow cytometry

For sub-G1 DNA content analysis, cells were trypsinized at the indicated time points and fixed in 80% ethanol at 4°C for a minimum of 30 minutes. Fixed cells were then stained with propidium iodide in the presence of RNase and analyzed on an Epics Elite flow cytometer (Beckman Coulter). For Ki-67 expression, cells were ethanol fixed, as described above, and immunostained with an anti-Ki-67 antibody (Sigma) at 1∶500 for 30 minutes. Cells were subsequently incubated with Alexafluor 488-conjugated antibody at 1∶500 in PBS for 30 minutes and resuspended in PBS for analysis.

### Western blot analysis

Cells were treated in log-phase growth, harvested by cell scraping, centrifuged, and lysed on ice for 2 hours with cell-lysis buffer. The supernatant was collected following centrifugation, and protein concentration was determined using the Bio-Rad protein assay (Bio-Rad Laboratories). Samples were electrophoresed under reducing conditions on NuPAGE 4–12% Bis-Tris gels (Invitrogen), transferred to PVDF, and blocked in 10% non-fat milk in TBST for 1 hour. Membranes were then incubated with primary antibodies obtained from Cell Signaling at 1∶1000 in 2% non-fat milk in TBST overnight at 4°C. Membranes were washed in TBST, incubated with the appropriate HRP-conjugated secondary antibody (Thermo-Scientific) for 1 hour, washed in TBST, and visualized using ECL-Plus (Amersham) and X-Ray film (Thermo-Scientific).

### *In vivo* studies

Athymic female nude mice (Charles River Laboratories) were inoculated with 1×10^6^ of 5-FU- resistant RKO or HT-29 cells in each rear flank as a 200 µL suspension of 1∶1 Matrigel (BD):PBS. Treatment was initiated once tumors reached a mean volume of ∼1650 mm^3^, intraperitoneal or intravenous injections were given at a total volume of 200 µL in DMSO. For tissue analysis, tissue was harvested from euthanized mice and fixed in 4% paraformaldehyde in PBS for 48 hours. Tissue was paraffin-embedded and sectioned by the Histology Core Facility at Penn State Hershey Medical Center. H&E staining (Daiko) and TUNEL staining (Millipore) were carried out according to the manufacturer's protocols. For serum chemistry assays, 1 mL of blood was harvested from anesthetized mice by terminal cardiac puncture of the left ventricle. For serum chemistry, 500 µL was placed into a microfuge tube and allowed to clot for 30 minutes at room temperature followed by centrifugation. Serum was removed, centrifuged again to remove any additional debris, and submitted for analysis by the Comparative Medicine Diagnostic Lab at Penn State Hershey Medical Center. All animal experiments were conducted in accordance with a protocol approved by the Institutional Animal Care and Use Committee (IACUC) at Penn State Hershey Medical Center.

### Statistics

Pairwise comparisons were assessed by the Student's two-tailed t-test in Microsoft Excel. Combination indices were computed with CalcuSyn software (BioSoft) using the Chou-Talalay method [8].

## Results

### Defining the ISC-4 *in vitro* activity profile

**Figure 1:**
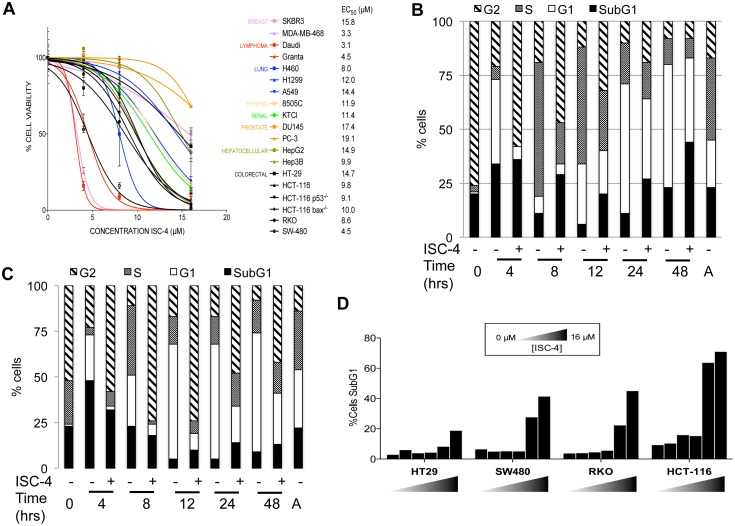
Monoagent ISC-4 activity *in vitro*. (A) Cell viability assays and calculated EC_50_ values for indicated cell lines treated with ISC-4 or DMSO (72 hr, n = 3). Effect of ISC-4 treatment on cell cycle profiles of synchronous and asynchronous (B) HCT116 and (C) HT-29 cell lines. Asynchronous cells are indicated by A in the figure. (D) Sub-G1 content of indicated colon cancer cell lines following ISC-4 treatment (0, 1, 2, 4, 8, or 16 µM).

We tested the mono-agent *in vitro* activity of ISC-4 in a panel of human cancer cell lines to characterize its spectrum of activity. Among the tested cell lines, the human lymphoma cell lines Daudi and Granta were the most sensitive, and the human prostate cancer cell lines PC3 and DU145 were the least sensitive in terms of EC_50_ values (Fig. 1A). With the exception of HT-29, human colon cancer cell lines were moderately sensitive to ISC-4 treatment. The isogenic HCT116 cell lines indicate that ISC-4 activity is likely p53- and Bax-independent. In order to better characterize the therapeutic effects of ISC-4 and potentially improve its combinatorial activity, we focused on the study of ISC-4 in colon cancer because of the moderate mono-agent activity [5].

Cell cycle analysis of ISC-4-treated synchronized HCT116 and HT-29 human colon cancer cell lines revealed a modest increase in sub-G1 content and a decrease in the rate of cell cycle progression (Fig. 1B–C). ISC-4-induced sub-G1 content is dose-dependent and generally becomes apparent at >8 µM (Fig. 1D). Thus, ISC-4 has modest single agent activity against human colon cancer cells by causing cell death and a decrease in proliferation.

### Identifying a synergistic combination with ISC-4

**Figure 2:**
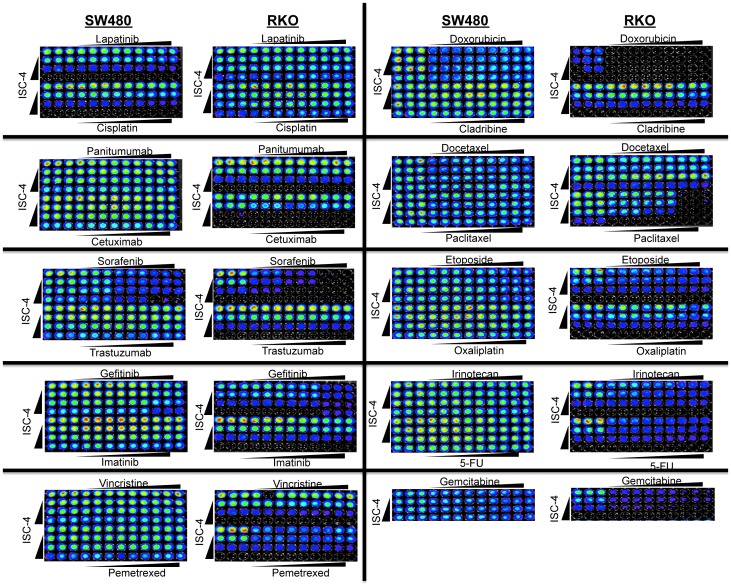
Combinatorial activity of ISC-4 and FDA-approved cancer therapies. Cell viability assays in SW480 and RKO colon cancer cell lines treated with ISC-4 (1, 2, or 4 µM) and indicated therapies at putative EC_12.5_, EC_25_, and EC_50_ alone and in combination (n = 3). Doses are provided in Table S1.

We searched for synergistic combinations of ISC-4 with FDA-approved therapies to improve the efficacy of ISC-4 against colon cancer. We selected the SW480 and RKO human colon cancer cell lines for initial profiling based on their heterogeneous oncogenic genetic alterations. SW480 has mutant *p53*, mutant *KRAS*, and wild-type *BRAF* whereas RKO has wild-type *p53*, wild-type *KRAS*, and mutant *BRAF* genes [9]. Among the test panel of chemotherapies and targeted agents, combinatorial activity was observed in at least one cell line when ISC-4 was combined with sorafenib, gefitinib, gemcitabine, cisplatin, bortezomib, imatinib, or cetuximab (Figure 2; Tables S1, S2). However, the combination of ISC-4 and cetuximab was the only synergistic combinatorial therapy observed under the tested conditions. Furthermore, this synergy was only observed in the RKO cell line, which harbors wild-type *KRAS*, and not in the SW480 cell line that harbors *KRAS^G12V^*. This observation is in accordance with the requirement of wild-type *KRAS* for the clinical efficacy of cetuximab in colon cancer [10], [11].

### ISC-4 and cetuximab synergistically inhibit wild-type KRAS tumor cell proliferation

**Figure 3:**
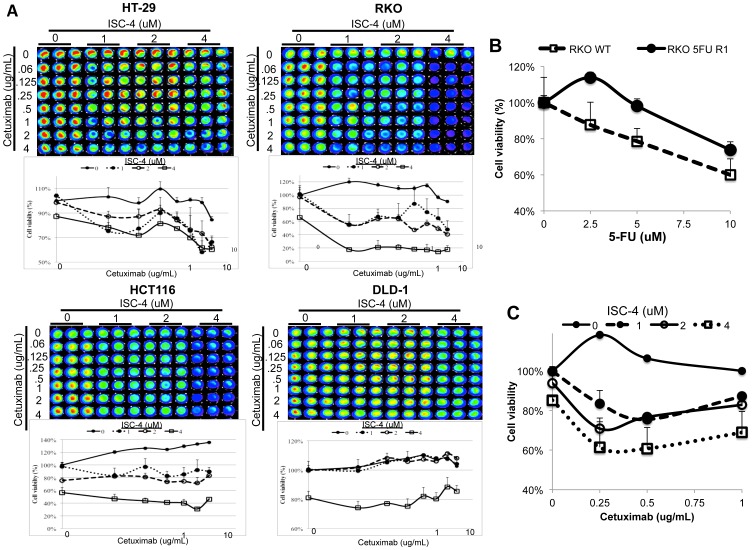
ISC-4 and cetuximab synergize in human colon cancer cells with wild-type *KRAS* genes independently of 5-FU sensitivity. (A) Cell viability assays of human colon cancer cell lines treated with ISC-4 and cetuximab at indicated doses for 72 hours (n = 3). (B) Cell viability assay of wild-type and 5-FU-resistant RKO cells treated with 5-FU as indicated for 24 hours (n = 3). (C) 5-FU-resistant RKO cells treated with ISC-4 (2 µM) and cetuximab (1 µg/mL) for 24 hours (n = 3).

We evaluated the synergistic activity of ISC-4 and cetuximab in several human colon cancer cell lines. In line with our previous observation, synergistic activity was only observed in HT-29 and RKO cell lines, which have wild-type *KRAS* genes, and not in HCT116, DLD-1, and other colon cancer cell lines with mutant *KRAS* genes (Fig. 3A; Table S3; data not shown). Interestingly, the synergistic interaction between ISC-4 and cetuximab appears to be dose-dependent with respect to ISC-4 concentration but independent of cetuximab concentration. To evaluate this combinatorial activity in a clinically relevant setting, we tested the synergistic efficacy of ISC-4 and cetuximab in RKO clones with evolved resistance to 5-FU. Because 5-FU is a standard-of-care therapy for the treatment of colon cancer, therapies that offer benefit in 5-FU-resistant colon cancer are needed in the clinic. The synergistic activity of ISC-4 and cetuximab was retained despite acquired 5-FU-resistance in the tested colon cancer cells (Fig. 3B–C). Together, these data identify 5-FU-resistant colon cancer with wild-type *KRAS* genes as a suitable clinical setting that may benefit from ISC-4 and cetuximab combinatorial therapy.

**Figure 4:**
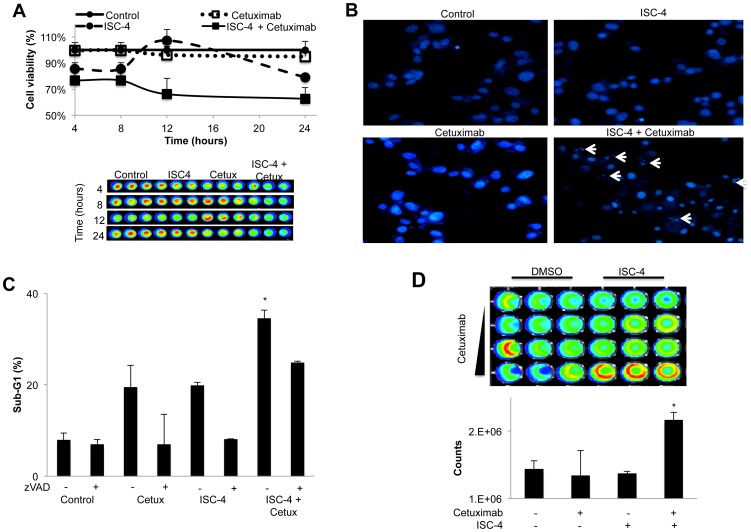
ISC-4 and cetuximab are cooperatively cytotoxic in 5-FU-resistant colon cancer cells. (A) Cell viability assays of RKO cells treated with ISC-4 (2 µM) and cetuximab (1 µg/mL) alone or in combination for the indicated time period (n = 3). (B) DAPI staining of RKO cells treated as in (A) for 12 hours. White arrows indicate cells with fragmented DNA. (C) Sub-G1 content of RKO cells treated with ISC-4 (2 µM) and cetuximab (1 µg/mL) alone or in combination for 12 hours (n = 3). *P<0.05 compared to all treatment groups by Student's two-tailed *t* test. (D) Caspase-Glo assay of RKO cells treated with ISC-4 (2 uM) in combination with cetuximab (0, 0.25, 0.5, or 1 µg/mL) at 24 hours post-treatment. Bottom panel shows quantification of ISC-4 (2 uM) and cetuximab (1 µg/mL) (n = 3).

We determined the kinetics of the observed synergistic efficacy and found such activity as early as 8 hours post-treatment, with greater synergy at 12 hours (Fig. 4A). This observation suggests that the synergistic activity of ISC-4 and cetuximab is perhaps cytotoxic rather than cytostatic. Changes in cell morphology, as well as fluorescent labeling of DNA, of treated cancer cells revealed that the ISC-4 and cetuximab combination treatment causes apparent DNA fragmentation (Fig. 4B; Fig. S1A). Further analysis revealed that the combination of ISC-4 and cetuximab cooperatively and significantly increase sub-G1 content compared to the mono-agents, but the combinatorial sub-G1 content was not sufficient to fully explain the observed synergy (Fig. 4C). ISC-4-induced sub-G1 content was significantly inhibited by co-incubation with the pan-caspase inhibitor zVAD-fmk, suggesting that the combination induces caspase-dependent apoptosis. In the support of this observation, the combination of ISC-4 and cetuximab synergistically induced caspase-3 activation (Fig. 4D).

**Figure 5:**
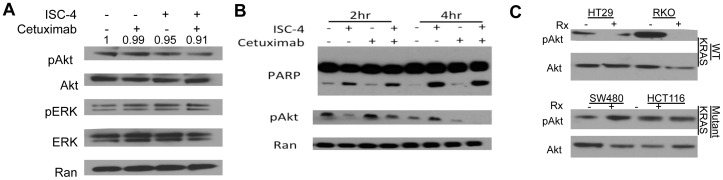
ISC-4 and cetuximab cooperatively reduce phospho-Akt. (A) Western blot analysis of RKO cells treated with ISC-4 (2 µM) and cetuximab (1 µg/mL) alone or in combination for 24 hours. Densitometry of phospho-Akt levels are shown above the blot as normalized to Ran as relative to control treatment. (B) Western blot analysis of RKO cells treated with ISC-4 (2 µM) and cetuximab (1 µg/mL) alone or in combination for indicated time periods. Ran is shown as a loading control. (C) Western blot analysis of indicated human colon cancer cell lines following treatment with the combination (Rx) of ISC-4 (2 µM) and cetuximab (1 µg/mL) for 8 hrs. *P<0.05 compared to control.

Western blot analysis revealed that ISC-4 in combination with cetuximab cooperatively reduces phospho-Akt levels, but not phospho-ERK, to a very modest level at 24 hours post-treatment (Fig. 5A). However, a time course analysis revealed that the combination cooperatively ablated phosho-Akt levels as soon as 4 hours post-treatment (Fig. 5B). Human colon cancer cell lines that exhibited a synergistic response to ISC-4 and cetuximab also responded with a significant decrease in phospho-Akt (Fig. 5C). However, human colon cancer cell lines harboring mutant KRAS that did not respond synergistically to the combination therapy also did not exhibit any changes in phospho-Akt levels in response to treatment. Thus, phospho-Akt levels appear to correlate with the antitumor response to ISC-4 and cetuximab. No effect on Ki-67 expression or LC3B cleavage, a marker of autophagy, was observed with the combination (Fig. S1B–D). These observations indicate that combing ISC-4 with cetuximab leads to a cooperative decrease in phospho-Akt and cell viability, which are accompanied by increased apoptosis.

### ISC-4 and cetuximab exert synergistic anti-tumor effects without toxicity *in vivo*

**Figure 6:**
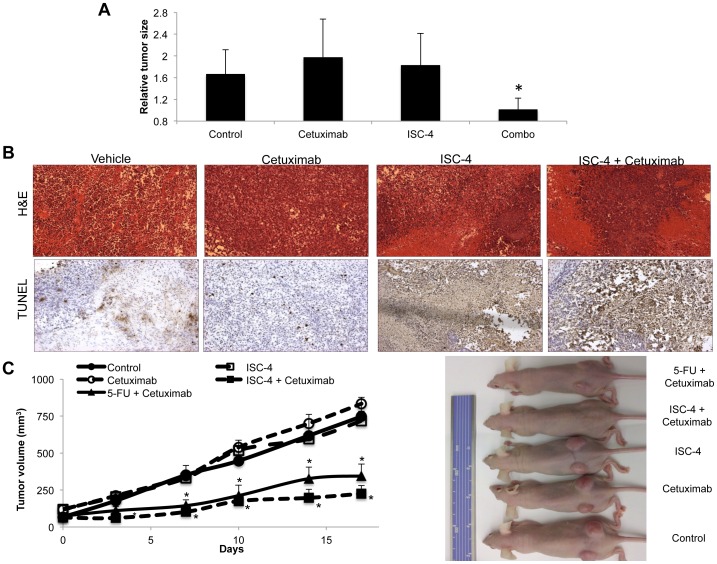
The ISC-4 and cetuximab has synergistic anti-tumor effects against advanced 5-FU-resistant colon cancer xenografts. (A) Relative tumor sizes of 5-FU-resistant RKO xenografts at 4 days post-treatment with a single dose of ISC-4 (3 mg/kg, i.p.), cetuximab (10 mg/kg, i.v.), or the combination (n≥5). Individual tumors were normalized to their baseline size measured on day 0. *P<0.05 compared to all treated groups using Student's two-tailed *t* test. (B) Hematoxylin and eosin (H&E) staining and TUNEL staining of xenograft tumors harvested 24 hours after treatment. (C) Athymic female nude mice harboring established HT-29 xenograft tumors were treated with ISC-4 (3 mg/kg, i.v.), cetuximab (10 mg/kg, i.v.), the combination, or cetuximab and 5-FU (25 mg/kg, i.v.) once per week starting on day 0 (n≥8). Error bars indicate SEM of replicates. *P<0.05 compared to control.

In order to mimic its likely clinical setting and utility, we tested the anti-tumor efficacy of ISC-4 in combination with cetuximab in advanced 5-FU-resistant RKO subcutaneous xenografts. In this setting, we found that the combination therapy has a synergistic initial effect on tumor progression and caused tumor stasis for the first week of therapy (Fig. 6A). Tissue analysis revealed that xenografts receiving the combination therapy had higher levels of necrosis by histology and apoptosis by TUNEL staining than xenografts receiving either agent alone (Fig. 6B; Fig. S2A). The therapeutic dosing regimen employed in these studies was well tolerated and did not alter mouse body weight or change in liver histology (Fig. S2B–C).

We next explored the combination of ISC-4 and cetuximab in HT-29 xenografts in mice in comparison with monoagents and the combination of cetuximab and 5-FU. We found that ISC-4 and cetuximab strongly reduced tumor progression, unlike the monoagents, when given as weekly intravenous doses that was grossly apparent by tumor volume and tumor weight measurements (Fig. 6C; Fig. S2D). Furthermore, the combination exhibited superior antitumor activity compared to the combination of 5-FU and cetuximab under these experimental conditions. This combination was again well tolerated (Fig. S2E) and furthermore, serum chemistry analysis revealed no significant changes in electrolytes, liver function, or other molecular markers related to kidney or cardiac toxicity with chronic dosing (Table S4). Cumulatively, these efficacy and safety data indicate that the combinatorial activity of cetuximab and ISC-4 should be evaluated in future clinical trials with 5-FU-refractory colon cancer harboring wild-type *KRAS* genes.

## Discussion

ISC-4 is a promising Akt inhibitor that has demonstrated anti-tumor activity in several preclinical studies [3], [4], [5]. The observed synergy between ISC-4 and cetuximab as a combination therapy allows ISC-4 to exert cytotoxicity at low micromolar doses against human colon cancer cells, which may be a more achievable dose *in vivo*. The combination appears to induce increased levels of apoptosis both *in vitro* and *in vivo*, though other anti-tumor mechanisms may contribute to the synergy. This observation is in line with previous finding that perifosine, a PI3K/Akt inhibitor, synergizes with agents that inhibit EGFR, such as cetuximab [12]. Future studies should compare ISC-4 with perifosine alone and in combination with cetuximab to determine their relative potency and potential as new treatments for human cancer.

Future studies should examine underlying mechanisms of synergy between the two agents. Special attention should be paid to the combinatorial effect on phospho-Akt, which serves as therapeutic response marker to the combination and may be employed in future clinical trials. Interestingly, the synergy between these two agents was observed in HT29 and RKO cells, which possess mutant BRAF and PIK3CA genes that should positively impact on Akt activity. Combinatorial *in vitro* activity was observed for ISC-4 with several other approved targeted agents and chemotherapies, although not synergistic, and merits further investigation. The activity of ISC-4 against lymphoma should be further explored given the relatively potent *in vitro* activity for single-agent ISC-4 that we observed against lymphoma cell lines. The p53-independent activity of ISC-4 and the conservation of synergy with cetuximab in 5-FU-refractory disease bode well for the clinical utility of ISC-4. Several chemotherapies, including 5-FU, have p53-dependent cytotoxic effects on tumor cell, and, therefore, chemotherapy-resistant disease commonly arises during disease progression due to frequent inactivation of the tumor suppressor p53 [13].

In addition to the tumor stasis induced by ISC-4 and cetuximab combination therapy in 5-FU-resistant tumors, it should be noted that the therapeutic dosing regimen was very well tolerated. This bodes well for its use in patients with advanced disease, many of whom are unable to tolerate harsh treatments. The therapeutic activity in 5-FU-resistant advanced colon cancer is promising and may benefit from the addition of other commonly used therapies in the context of colon cancer management, such as oxaliplatin. Cetuximab has been shown to restore oxaliplatin sensitivity in refractory colon cancer cells and may represent a promising therapeutic opportunity [14]. Clinical data also support the combination of cetuximab with irinotecan in irinotecan-refractory disease [15].

Given the demonstrated efficacy and safety of this combination, clinical investigation of ISC-4 in combination with cetuximab is warranted in patients with wild-type *KRAS* genes, as is required for standard cetuximab therapy [11]. Recent evidence suggests that a biopsy should be taken immediately prior to treatment initiation to assure KRAS is wild-type as colon cancer patients can evolve KRAS mutations during cetuximab treatment that causes resistance [16]. Furthermore implementation of phospho-Akt as a biomarker of response may prove useful in the future trial through tumor biopsies or analysis of circulating tumor cells [17], [18]. This preclinical study argues that the combination may offer a safe therapeutic benefit in the face of 5-FU resistance for colon cancer patients that need more treatment options.

## Supporting Information

Figure S1 **ISC-4 and cetuximab combination therapy does not decrease Ki-67 expression or induce autophagy.** (A) Phase-contrast microscopy of RKO cells treated with ISC-4 (2 µM) and cetuximab (1 µg/mL) alone or in combination for 12 hours. (B) (B) Flow cytometry and (C) Western bot analysis of Ki-67 expression in RKO cells treated with ISC-4 (2 µM) and cetuximab (1 µg/mL) alone or in combination as determined by. (D) Western blot analysis of RKO cells treated with ISC-4 (2 µM) and cetuximab (1 µg/mL) alone or in combination for 24 hours. Chloroquine (C; 10 µM) is included as a positive control for autophagy. Beta actin is shown as a loading control.
(TIF)

Figure S2 **ISC-4 and cetuximab combination therapy is safe and exerts cooperative antitumor activity.** (A) Quantification of TUNEL staining in tumor xenografts described in Figure 6B (n = 10). (B) Change in body weight of mice receiving ISC-4 (3 mg/kg, i.p.), cetuximab (10 mg/kg, i.v.), or the combination (n≥5) twice a week for 2 weeks. Body weight changes are expressed relative to the body weight of each individual mouse prior to treatment on day 0 (n≥3). (C) H&E staining of liver tissue harvested from mice at 24 hours post-treatment with ISC-4 (3 mg/kg, i.p.), cetuximab (10 mg/kg, i.v.), or the combination. (D) Terminal tumor volume and tumor weight for HT-29 xenograft described in Figure 6C. Treatment cohorts included ISC-4 (3 mg/kg, i.v.), cetuximab (10 mg/kg, i.v.), the combination, or cetuximab and 5-FU (25 mg/kg, i.v.) once per week (n≥8). (E) Mouse body weight at endpoint, which was three days following the last dose (n≥8). Error bars indicate SEM of replicates.
(TIF)

Table S1 **Doses selected for approved antitumor agents in combination with ISC-4.** EC_12.5_, EC_25_, and EC_50_ values were estimated from the literature and doses were employed in experiments described in Fig. 2.
(XLSX)

Table S2 **Summary of combinatorial effects of TIC10 with approved antitumor agents.** Combinatorial activity were compared to monoagent activities by cell viability assays and determined to be uncooperative (−), cooperative (+), synergistic (*), or ambiguous (?). Combinations exhibited cooperative activity in at least one cell line are highlighted in yellow whereas the green highlight indicates synergy.
(XLSX)

Table S3 **Combination indices for the ISC-4 and cetuximab in wild-type KRAS human colon cancer cell lines.** Combinatorial activity in RKO and HT-29 cell lines quantified in Figure 3A was assessed by the Chou-Talalay method.
(XLSX)

Table S4 **Serum chemistry profiles of mice receiving ISC-4 and cetuximab combination therapy.** Athymic, female 8-week old nude mice received ISC-4 (3 mg/kg, i.p.), cetuximab (10 mg/kg, i.v.), or the combination (n≥5) twice a week for 2 weeks. Serum was collected 2 days following the last dose.
(XLSX)
