# Correction: The Akt Inhibitor ISC-4 Synergizes with Cetuximab in 5-FU-Resistant Colon Cancer

**DOI:** 10.1371/annotation/aaa12360-0c90-42c3-bc43-d00022b68b81

**Published:** 2013-11-07

**Authors:** Joshua E. Allen, Jean-Nicolas Gallant, David T. Dicker, Shantu Amin, Rosalyn B. Irby, Arun K. Sharma, Wafik S. El-Deiry

There is an error in the title of Supplemental Table S2. The table describes the combinatorial effects of ISC-4, not TIC10. The correct title reads: Summary of combinatorial effects of ISC-4 with approved antitumor agents. 

