# Supplementary figures and images for: The Akt Inhibitor ISC-4 Synergizes with Cetuximab in 5-FU-Resistant Colon Cancer
Source: PLoS One. 2013 Mar 12;8(3):e59380. doi: 10.1371/journal.pone.0059380 (PMC3595267; doi:10.1371/journal.pone.0059380)

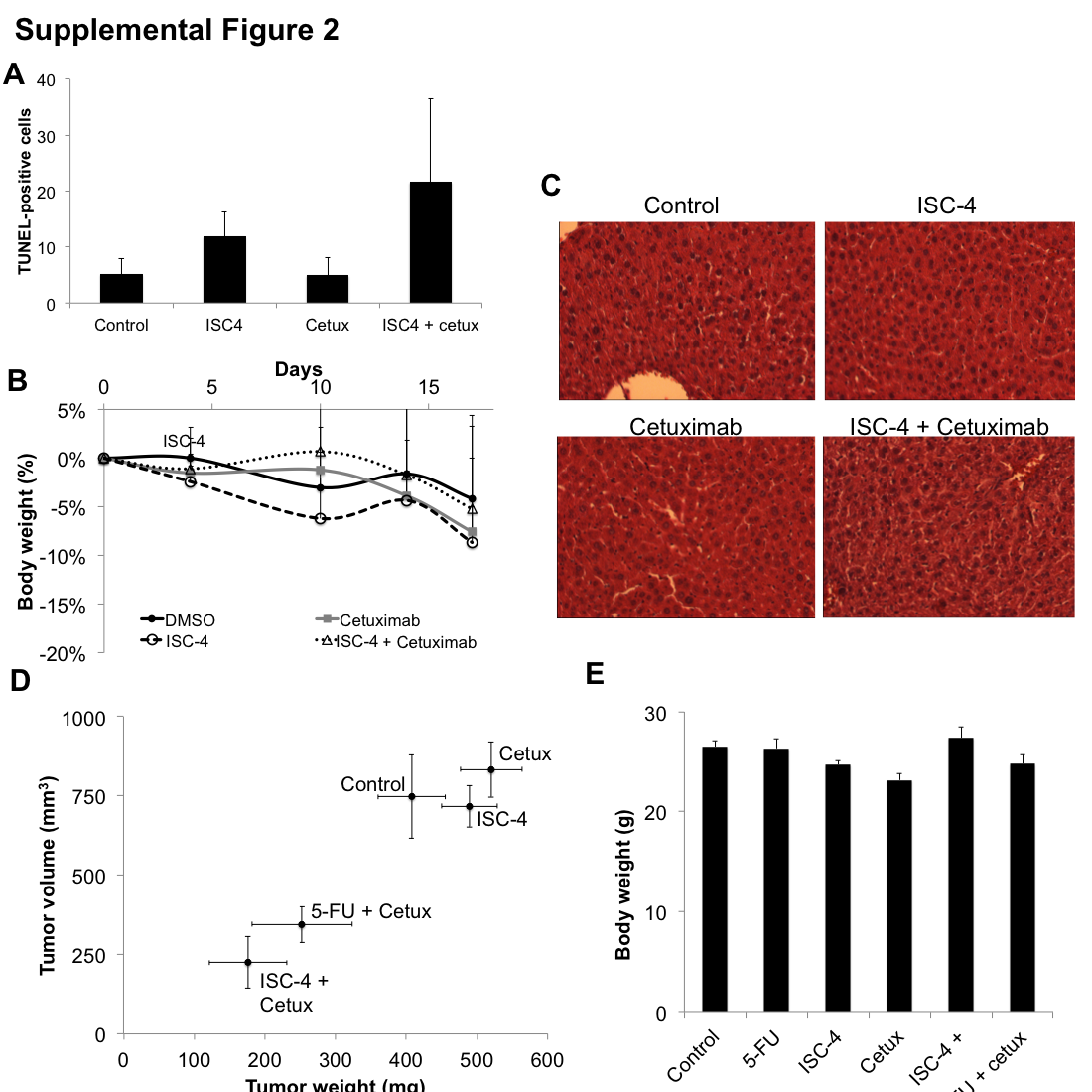

Supplement: Figure S2 — ISC-4 and cetuximab combination therapy is safe and exerts cooperative antitumor activity. (A) Quantification of TUNEL staining in tumor xenografts described in Figure 6B (n = 10). (B) Change in body weight of mice receiving ISC-4 (3 mg/kg, i.p.), cetuximab (10 mg/kg, i.v.), or the combination (n≥5) twice a week for 2 weeks. Body weight changes are expressed relative to the body weight of each individual mouse prior to treatment on day 0 (n≥3). (C) H&E staining of liver tissue harvested from mice at 24 hours post-treatment with ISC-4 (3 mg/kg, i.p.), cetuximab (10 mg/kg, i.v.), or the combination. (D) Terminal tumor volume and tumor weight for HT-29 xenograft described in Figure 6C. Treatment cohorts included ISC-4 (3 mg/kg, i.v.), cetuximab (10 mg/kg, i.v.), the combination, or cetuximab and 5-FU (25 mg/kg, i.v.) once per week (n≥8). (E) Mouse body weight at endpoint, which was three days following the last dose (n≥8). Error bars indicate SEM of replicates. (TIF) [file pone.0059380.s002.tif]
